# Neglected increases in rural road traffic mortality in China: findings based on health data from 2005 to 2010

**DOI:** 10.1186/1471-2458-13-1111

**Published:** 2013-12-01

**Authors:** Yuanxiu Huang, Danping Tian, Lin Gao, Li Li, Xin Deng, Keita Mamady, Guoqing Hu

**Affiliations:** 1 Department of Epidemiology and Health Statistics, School of Public Health, Central South University, Changsha, China

**Keywords:** Road traffic mortality, Urban areas, Rural areas, Motor vehicle crash, China

## Abstract

**Background:**

Recent changes in rural road traffic mortality have not been examined in China although rural residents were reported as having greater risk of road traffic injury than urban residents. We aimed to examine changes in urban and rural road traffic mortality rates between 2005 and 2010 in China.

**Methods:**

Mortality rates came from the publicly available health data of the Ministry of Health-Vital Registration System that is based on a national representative sample (about 10% of total population), including 41 surveillance points in urban areas (15 large cities and 21 middle/small cities) and 85 surveillance points in rural areas. The causes of deaths were coded using the Tenth International Classification of Diseases (ICD-10). Linear regression was used to test the statistical significance of changes in mortality rates. We calculated the percent change in rates to quantify the change between 2005 and 2010, which was calculated as regression coefficient * 100 * 5 divided by the rate in 2005.

**Results:**

In rural areas, road traffic mortality increased by 70%, changing from 13.3 per 100,000 population in 2005 to 22.7 per 100,000 population in 2010. In contrast, the road traffic mortality merely increased by 4% in the study time period, rising from 13.1 to 13.9 per 100,000 population in urban areas. Both the increases in road traffic mortality from motor vehicle crashes and from non-motor vehicle crashes were larger in rural areas than in urban areas (106% vs. 4%; 29% vs. 3%).

**Conclusion:**

The tremendous increase in road traffic mortality in rural areas calls for urgent actions to reduce road traffic injuries to motor vehicle occupants, motorcyclists, bicyclists and pedestrians in in rural areas.

## Background

Road traffic crashes caused 1.3 million global deaths in 2010 [1]. According to the *Global status report on road safety: time for action*[2], China was estimated to account for 18% of global road traffic deaths. China has implemented many control measures to curb the rising threat from road traffic crashes in the last two decades, such as setting speed limits, standardizing warning signs, improving driver licensing, and inducing severe punishment for law violations [3,4]. Many efforts, however, are obviously weaker in rural areas than in urban areas due to the limitation of resources, like the improvement of law enforcement and road infrastructure [5].

Urban–rural differences in road traffic injury were not completely consistent in previously publications. Urban areas were reported to have high road traffic mortality in India [6,7] and sustain more injuries in South East Iran [8] compared rural areas. In Nigeria, the difference in road traffic incidence in the past 12 months between urban areas and rural areas was insignificant [9]. While in China [5,10], South Africa [11], and USA [12], rural areas were reported having higher road traffic mortality than urban areas.

While rural residents were reported as having greater risk of road traffic injury than urban residents in China [5,10], the recent change in rural road traffic mortality has not been examined so far. This is primarily because that the relevant information on rural areas is excluded from the publicized police data (combined data) -- the most cited data source of road traffic injury. The lack of rural data may provide a misconception that rural areas have experienced equal changes in road traffic mortality as urban areas recently and no policy changes are needed to improve rural road safety.

To attain the objectives of the Decade of Action for Road Safety 2011–2020, it is important to provide the information of recent changes in rural road traffic mortality. We examined the changes in road traffic mortality rates of 2005–2010 by using the Ministry of Health-Vital Registration (MOH-VR) aggregate data.

In terms of large gap in the prevention efforts to road traffic injuries between urban areas and rural areas, we assumed that rural areas experienced larger increase or smaller decrease in road traffic mortality than urban areas between 2005 and 2010.

## Methods

### Data source

There are four national-level data sources involving road traffic injury, including Ministry of Health-Vital Registration (MOH-VR) System, Chinese CDC-Disease Surveillance Points (DSP), Chinese CDC-National Injury Surveillance System (NISS), and police reports [13]. Currently, road traffic mortality rates on urban areas and rural areas can be freely accessed only for the MOH-VR aggregate data that are published in the Chinese Health Statistics Yearbook.

In China, a six-class classification of urbanization is used according to the number of population: (1) megacity having >1 million population; (2) large city having 0.5-1 million population; (3) medium city having 0.2-0.5 million population; (4) small city having 20,000 to 0.2 million population; (5) town having 2,000 to 20,000 population; and (6) village having <2,000 population [14].

The surveillance points of MOH-VR includes three categories: (1) large cities that have >0.5 million population; (2) medium and small cities that have population falling between 20,000 and 0.5 million; and (3) rural areas that have <20,000 population [15]. The MOH-VR system currently covers 10% of the total population including 41 urban centers (15 large cities and 21 middle/small cities) and 85 rural centers [13].

The data are collected based on physicians’ death certificates, which are submitted to local police departments by family members and then forwarded to the municipal, provincial, and national departments of health. Families are requested to provide death certificates to get permission for cremation or burial [16]. The causes of deaths are based on physicians’ death certificates and are coded using the Tenth International Classification of Diseases (ICD-10) since 2002.

### Statistical analysis

We used linear regression to examine the significance of changes in mortality rates in the study period. The percent change in rates was used to measure the change between 2005 and 2010, which was calculated as regression coefficient * 100 * 5 divided by the rate in 2005. The formula takes the fluctuations in the intervening years into account by fitting the linear regression between death rate and year in this period. An alpha of p < 0.05 was considered statistically significant. SPSS18.0 was used for data analysis.

## Results

Between 2005 and 2010, total road traffic mortality increased by 70% in rural areas (from 13.3 to 22.7 per 100,000 persons), while the change was 4% in urban areas, yielding an enlarged urban–rural gap (Table 1). Subgroup analysis by place and crash type showed that (1) in contrast to the small change (4%) in urban areas, the mortality rate from motor vehicle crashes doubled from 2005 to 2010 in rural areas; and (2) the increase in non-motorized-vehicle traffic mortality rates in rural areas was also larger than in urban areas (29% vs. 3%) although both were not statistically significant (*p* > 0.05).

**Table 1:** Age-adjusted mortality from road traffic crashes (China, 2005–2010)

**Place/crash type**	**2005**	**2006**	**2007**	**2008**	**2009**	**2010**	**Percent change in rates**
Urban areas							
All road traffic crashes	13.1	11.5	13.7	10.7	12.4	13.9	4
Motor vehicle crashes	9.1	6.2	8.2	6.9	8.4	8.7	4
Non-motorized-vehicle crashes	4.0	5.3	5.5	3.8	4.1	5.2	3
Rural areas							
All road traffic crashes	13.3	16.6	19.2	19.2	22.8	22.7	70^**^
Motor vehicle crashes	7.1	11.3	11.4	10.9	15.4	15.3	106^*^
Non-motor-vehicle crashes	6.2	5.3	7.8	8.3	7.4	7.4	29

*: *p* < 0.05; **: *p* < 0.01.

Percent change in rates was calculated as regression coefficient * 100 * 5 divided by the rate in 2005.

## Discussion

Between 2005 and 2010, rural residents experienced larger increase in road traffic mortality than urban residents for both motor vehicle crashes and non-motor vehicle crashes. The larger increases in rural areas may mainly reflect the combined effects of accelerated motorization, lack of road safety infrastructure [17], under-developed pre-hospital aids and hospital emergency treatment [18], and relatively high traffic law violations [15] in rural areas.

Although the statistics of motor vehicles are lacking for rural areas, the fact that total civil motor vehicles increased from 21.3 million in 2005 to 61.2 million in 2010 [19] indicates rapid motorization in rural areas to some extent. Compared to urban areas, road safety infrastructure is seriously lacking in rural areas. For example, almost all rural roads have no sidewalks, bicycle lanes, traffic lights, and speed-recording cameras [17]. In China, the pre-hospital aids and hospital emergency treatment in rural areas obviously lag behind that in urban areas [18].

In addition, because of the lack of road traffic safety control measures, rural areas have higher traffic law violations than urban areas, such as drunk driving and speeding [15].

Clearly, to curb the growing road traffic mortality in rural areas is critical for the success of the Decade of Action for Road Safety 2011–2020 in China. To attain the objective, China needs to do more to improve the road traffic safety in rural areas. First, the enforcement of traffic safety laws should be enhanced in rural areas, such as road traffic crashes from speeding, drink driving, drowsy driving and distracted driving. Second, the government should improve road safety infrastructures in rural areas such as traffic lights, sidewalks, cycle tracks and speed cameras, when finances allow.

Our findings were mainly limited by the lack of non-fatal injury data and information of covariates such as alcohol use, driving speed, characteristics of crash location and motor vehicles, and personal walking behaviors. The absence of related information prevents from quantitatively determining specific factors that contribute to larger increases in road traffic mortality in rural areas. In addition, the quality of MOH-VR data may have a slight effect on our findings since the data may suffer from incompleteness and/or coding errors to some degree. Because the MOH-VR collection system has remained relatively stable and used the ICD-10 codes since 2002 [20], it can be assumed that the quality of data would not obviously affect the estimation of changes in road traffic mortality between 2005 and 2010.

## Conclusion

To conclude, the large increase in rural road traffic mortality merits the attention of researchers and policy-makers. Urgent actions need to be taken to curb the rising rural road traffic mortality.

## Abbreviations

ICD-10: The 10^th^ International Classification of Diseases; MOH-VR: The Ministry of Health-Vital Registration.

## Competing interests

We declare we have no competing interests.

## Authors' contributions

All authors participated in the design, data analysis and the writing of the paper. GH supervised the whole process and finalized the paper. All authors read and approved the final manuscript.

## Pre-publication history

The pre-publication history for this paper can be accessed here:

http://www.biomedcentral.com/1471-2458/13/1111/prepub
